# Lipid Metabolism in the Development and Progression of Vascular Cognitive Impairment: A Systematic Review

**DOI:** 10.3389/fneur.2021.709134

**Published:** 2021-11-19

**Authors:** Qi Qin, Yunsi Yin, Yi Xing, Xuan Wang, Yan Wang, Fan Wang, Yi Tang

**Affiliations:** ^1^Innovation Center for Neurological Disorders, Department of Neurology, Xuanwu Hospital, Capital Medical University, National Clinical Research Center for Geriatric Diseases, Beijing, China; ^2^Department of Endocrinology, Mudanjiang Second People's Hospital, Mudanjiang, China; ^3^Department of Epidemiology, School of Public Health, Harbin Medical University, Harbin, China; ^4^Neurodegenerative Laboratory of Ministry of Education of the People's Republic of China, Beijing Key Laboratory of Geriatric Cognitive Disorders, Beijing, China

**Keywords:** lipid metabolism, vascular cognitive impairment (VCI), brain lipids, diagnostic biomarkers, peripheral biomarkers

## Abstract

**Background:** Vascular cognitive impairment (VCI) is a major public health problem. The current diagnosis of VCI is made based on the assessment of clinical symptoms and neuropsychological measurements, and is supported by neuroimaging. These methods are both time-consuming and expensive, which leads to needs for alternative biomarkers for VCI. Metabolomics is an emerging and powerful tool to discover of new biomarkers of disease, which can investigate variations in different metabolic processes such as lipid, since the brain is highly enriched in lipids and that lipid changes may lead to pathology in the brain. Vascular cognitive impairment is vulnerable to the disturbance of lipid metabolism. Furthermore, blood samples, which could be identified as reliable clinical biomarkers are relatively convenient to obtain and provide a non-invasive assessment. Therefore, our study aims to understand whether peripheral lipid biomarkers can be used as diagnostic biomarkers and monitor the progression of VCI.

**Methods:** We systematically searched the PubMed, Embase, CNKI, and VIP databases to find VCI and lipid metabolism in reports from inception through February 2021. Studies meeting the following criteria were eligible: (1) original studies in humans; (2) lipid metabolites in blood; (3) reports of VCI.

**Results:** Through our review, nine original articles were eligible. Blood-based metabolites that might be potential biomarkers were identified. Most of them including PC, PE, Cers, and ChEs were significantly lower, while elevation of FAs and DGs were associated with VCI. Most importantly, these blood-based metabolites might be proposed as potential biomarkers for VCI, which provides direction for further validation.

**Discussion and Conclusion:** To the best of our knowledge, this is the first systemic review concerning the relationship of lipid metabolism and VCI. It identifies potential biomarkers and provides insights into the disease pathobiology. However, more advanced studies and researches on a lipidomic platform must be done to understand the exact pathology behind and identify potential lipid biomarkers, which might help achieve the goal of discovering novel therapeutics.

## Introduction

Vascular cognitive impairment (VCI) was first put forward by Bowler in 1995 and has been considered an “umbrella term” in describing the cognitive and behavioral changes that occurs as a result of many different vascular pathologies. It is a wide spectrum of cognitive and behavioral changes ranging from subjective cognitive decline to dementia in recent years (1). Subtypes of VCI are divided according to the level of cognitive decline, ranging from the impairment of a single cognitive domain (mild VCI) to overt vascular dementia (VaD) or mixed dementia (vascular and degenerative, most commonly of Alzheimer's type) (2). Vascular dementia is the second most common cause of cognitive decline, accounting for 15–30% of cases (3, 4), creating challenges for policy makers, health-care professionals, and family members. It can be caused by cerebrovascular disease of different etiologies, including stroke, subcortical ischaemic vascular disease (SIVD), multi-infarct strokes, border-zone ischaemia, and intracerebral hemorrhages (4). Therefore, post-stroke cognitive impairment (PSCI) and SIVD are considered two subtypes of VCI. Post-stroke cognitive impairment is particularly important and frequent, occurring in approximately one-third of all stroke patients and having a significant negative impact on rehabilitation outcomes, quality of life, and risk of dementia (5).

Metabolomics is an emerging and powerful tool to map global biochemical changes or to discover of new biomarkers of disease (6), since it focuses on the dynamic changes in endogenous metabolites an organism. Metabolomics studies could investigate variations in different metabolic processes such as lipid, energy, amino acid, purine, and neurotransmitter metabolism, and oxidative stress. Metabolic profiling has been successfully applied in various neuropsychiatric disorders such as Alzheimer's disease and schizophrenia (5). The brain is highly enriched in lipids that play essential roles in the central nervous system (CNS). Neuron store and transport a significant number of lipids including sphingolipids, cholesterol, phosphatidylglycerol (PG), and polyunsaturated fatty acids (FA), which have two major functions: as structural components of neuronal membranes and as metabolic signaling molecules and neuromodulators in the CNS (7, 8). Lipids in astrocytes, such as phosphatidic acid, are used for both metabolic and signaling processes (8). These lipids together maintain a delicate balance. When this delicate balance is disturbed, it can easily lead to pathology in the brain. Vascular cognitive impairment is frequently characterized by major changes in white matter (WM), a part of the brain very rich in lipids. The WM contains neural networks formed by bundles of axons. Myelin acts as electrical insulation for axons and is responsible for rapid salutatory impulse propagation and protects the nerve fibers from injury. Its dry mass is mainly composed of proteins (15–30%) and lipids (70–85%), with phospholipids, cholesterol, galactolipids, and plasmalogens in a molar ratio of 2:2:1:1 (9). Therefore, VCI can be vulnerable to the disturbance of lipid metabolism. Recently, numerous studies have indicated that lipid metabolism might be involved in the pathogenesis of VCI (10). However, it is uncertain how lipid metabolism is involved in VCI. No systematic review or meta-analysis has been published concerning the correlation between lipid metabolism and VCI.

Currently, the diagnosis of VCI is based on the assessment of clinical symptoms and neuropsychological measurements and supported by neuroimaging. These diagnostic methods are both time-consuming and financially demanding, which leads to the need for other biomarkers that can help us evaluate VCI without the aforementioned disadvantages. These biomarkers should be used as a large-scale screening tool to evaluate pathogenic processes, as well as the patient's pharmacologic responses to intervention. In addition, continuous development in analytical techniques provides feasible determination in complex samples (11), for example, in cerebrospinal fluid (CSF), blood, urine, or post-mortem tissue. Among them, blood samples are relatively convenient and fast to obtain and provide a non-invasive assessment approach. These easy-to-measure biomarkers will be applicable to many more people. Thus, if blood lipid metabolites could be identified as reliable clinical biomarkers, the diagnosis, and monitoring of VCI would be much easier. Therefore, our study focuses on the blood-based biomarkers of VCI patients.

This study will help us understand whether these peripheral lipid biomarkers can be used as diagnostic biomarkers and monitoring biomarkers of VCI, which is useful for screening in large-scale clinical trials and for future clinical usage. Furthermore, this systemic review aims to provide insights into the pathobiology of VCI, showing how lipid metabolism acts in the development and progression of VCI, which might help achieve the ultimate goal of discovering novel therapeutics.

## Methods

### Information Sources and Search Terms

The systematic search strictly followed the Cochrane Handbook of Systematic Reviews (https://training.cochrane.org/handbook) and used the PRISMA-ScR (Preferred Reporting Items for Systematic Reviews and Meta-Analyses extension for Scoping Reviews) checklist. Four databases, PubMed, Embase, CNKI and VIP were searched for papers. The 28st of February, 2021 was the latest date manuscripts were considered for the literature search. We used keywords such as “lipid”, “lipid metabolism,” “vascular cognitive impairment,” “vascular dementia,” “post-stroke dementia,” “post-stroke cognitive impairment” separately and in combination with each other. We also manually searched references of relevant articles and reviews during screening.

### Study Selection and Eligibility Criteria

All titles and abstracts were screened by two independent reviewers; disagreements between them were resolved by full-text review and consensus. Studies were considered eligible for inclusion based on the following criteria: (1) participants were VCI patients and controls (PSCI is also included because it is the most important and frequent subtype of VCI); (2) lipid metabolites in blood (plasma or serum) were detected by mass spectrometry (MS) based analytical platforms or other analytical techniques; and (3) original studies in humans (e.g., cross-sectional studies, cohort studies, case-cohort studies). Exclusion criteria were: (1) randomized controlled trials (RCTs) with interventions for patients; (2) articles not providing detailed information of the participants; (3) non-human studies; (4) metabolomics studies in CSF, saliva, and urine samples and those using magnetic resonance spectroscopy (MRS) for the quantification of brain metabolites.

### Data Extraction

Study characteristics, including first author, year of publication, study population and location, study design, mean age of participants, participant characteristics, sample size, biospecimens (plasma or serum), analytical technique and metabolite targets, outcome, statistical method, covariates included in the fully adjusted model, and a summary of key findings (analyzed metabolites and effect estimates), were extracted from each included study.

### Quality Assessment

The quality of the studies was assessed and scored independently by two researchers.

A modified Newcastle-Ottawa Scale was used in the assessment of case-control studies. Cross-sectional studies were evaluated by the Agency for Healthcare Research and Quality (AHRQ) tool. Details of these criteria and the score of each study are presented in Supplementary Tables 1, 2. Disagreements between reviewers in quality assessment were resolved through consensus.

## Results

### Literature Retrieval

**Figure 1:**
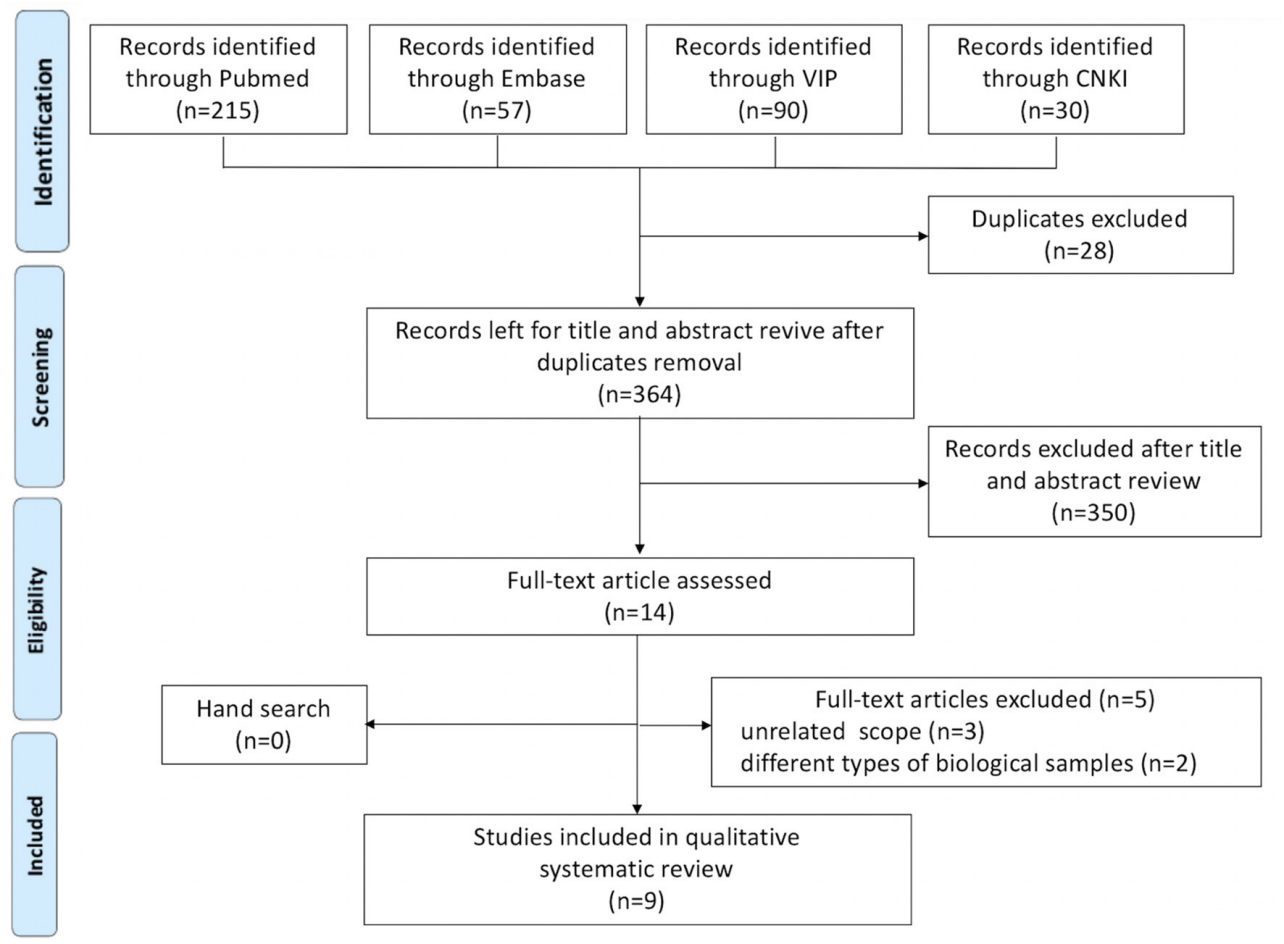
Flow diagram of literature search and study selection.

In the initial search, 364 unique studies were identified after removal of duplicates. Following the review of titles and abstracts, 14 articles were selected for careful full-text screening, nine were eligible (Figure 1). All these studies were excluded because of study design, unrelated scope, different types of biological samples, or use of animal rather than subjects, or because they were duplicates of included studies.

### Study Characteristics

The characteristics of the nine included studies are summarized in Table 1. Seven of the nine studies were conducted in China, while the others were conducted in Australia and Tunisia. Nearly half (4/9) of the studies were published after 2017. The total number of participants was 1,391, ranging from 80 to 360. Of the nine longitudinal analyses, seven studies used a cross-sectional study design, and the other two analyses were based on case-control studies.

**Table 1:** Characteristics of studies investigating associations between lipid metabolites and VCI.

**References**	**Study population**	**Study design**	***N***	**Biospecimen**	**Baseline cognition status of participants**	**Mean age**	**Outcome (N); ascertainment**	**Analytical platform, lipid metabolite targets**
Liu et al. (10)	Australian	Cross-sectional study	97	Fasting EDTA plasma	Healthy control (49); Vascular dementia (48)	>65	VaD patients were diagnosed using the criteria of NINCDS-AIREN	Liquid-chromatography coupled to mass spectrometry (LC/MS); 667 lipid species from 9 lipid subclasses including ChE, glycerolipids DG, TG, Cers, SMs,PC, PE, and PI.
Liu et al. (5)	Chinese	Cross-sectional study	80	Fasting serum	Healthy control (20); PSNCI (30); PSCI (30)	PSCI: 66.10 ± 6.50; PSNCI: 67.50 ± 8.64 Healthy: 67.30 ± 6.81	The cognitive status of the inpatients were measured using the Montreal cognitive assessment (MoCA); PSCI patients was defined as stroke patients with MoCA <24 and excluded dementia before stroke	Ultra-high performance liquid chromatography coupled with Q-TOF mass spectrometry (UHPLC-Q-TOFMS analysis); LysoPC (16:0), LysoPC (18:0) and palmitoylcarnitine, formic acid, carnitine, creatinine, glutamine, proline, citric acid, valine, hypoxanthine, uric acid, tyrosine, isoleucine, kynurenine, phenylalanine, and tryptophan
Zarrouk et al. (12)	Tunisia	Cross-sectional study	192	Fasting EDTA plasma; RBC	Healthy control (128); Demented patients (64)	Patients: 65 ± 10 Control: 72 ± 8	_______	Gas chromatography (GC) and gas chromatography coupled with mass spectrometry set to the selected-ion monitoring mode (GC/MS-SIM); fatty acids, VLCFAs (C22:0; C24:0; C26:0), phytanic acid, and plasmalogen-C16:0
Zhang et al. (13)	Chinese	Case control study	226	Serum	VD (104); VCIND (68); Stoke patients with normal cognition status (54)	VD: 66 ± 5 Other stroke patients: 69 ± 6	CSVD patients were diagnosed using the criteria of WHO; VD and VCIND was diagnosed using the criteria of DSM-V	ELASA; Lp-PLA2
Niu et al. (14)	Chinese	Cross-sectional study	360	Serum	Healthy control (60); CSVD patients with normal cognition status (106); CSVD patients with cognitive impairment (194)	Patients: 68.05 ± 5.65 Control: 67.03 ± 4.98	CSVD patients were diagnosed using the criteria of Chinese Consensus of CSVD (2015);VCI was diagnosed using the criteria of Chinese guideline of VCI (2016)	Double antibody sandwich-enzyme linked immunosorbent assay, DAS-ELISA; Lp-PLA2
Zhang et al. (15)	Chinese	Case control study	120	Serum	Healthy control (60); VCIND (60)	Patients: 73.6 ± 6.4 Control: 72.9 ± 8.1	VCIND patients were diagnosed using the criteria raised by Jia in 2004	PAFAH kit; Lp-PLA2 activity
Hou et al. (16)	Chinese	Cross-sectional study	120	Plasma	Control (42); VaD (40); AD (38)	VaD:70.1 ± 10.0 AD: 73.7 ± 8.2 Control: 71.2 ± 7.8	AD patients were diagnosed using the criteria of NINCDS-ADRDA; VaD patients were diagnosed using the criteria of NINCDS-AIREN	ELASA; Lp-PLA2
Bai et al. (17)	Chinese	Cross-sectional study	95	Plasma	Control (50); VCIND (45)	VaD: 66 ± 5 Control: 69 ± 6	_______	ELASA; Lp-PLA2
Bai and Su (18)	Chinese	Cross-sectional study	107	Plasma	Control (31); VCIND (47); VaD (29)	49–75	VCIND and VaD patients were diagnosed using the criteria raised by Rockwood in 1999	ELASA; Lp-PLA2

### Biological Samples and Analytical Platform

Five studies analyzed plasma and the other four studies used serum samples. ELISA was used in five studies, while the other four used liquid chromatograph/mass spectrometry (LC/MS), ultra-high performance liquid chromatography-quadrupole time-of-flight mass spectrometer (UHPLC-Q-TOFMS) analysis, gas chromatograph/mass spectrometry-selected-ion monitoring (GC/MS-SIM) and PAFAH kit, respectively. The majority of studies adopted targeted approaches, while untargeted metabolomics analysis was adopted in only three studies.

### Lipid Metabolism Associated With VCI

Concentrations of several blood lipid metabolites, including phospholipids, sphingolipids, and FA were associated with cognitive decline and the incidence or progression of VCI. Most of them including phosphatidylcholines (PC), phosphatidylethanolamines (PE), ceramide (Cer), and cholesterol esters (ChE) were significantly lower, while FA and diacylglycerol (DG) accumulated in plasma. The results of studies associating metabolites with VCI are described as follows and summarized in Table 2.

**Table 2:** Results of analyses associating lipid metabolites with VCI.

**References**	**Statistical analysis**	**Covariates in fully adjusted model**	**Statistically significant**	**Key finding**
Liu et al. (10)	Bonferroni correction; Random forests; Logistic regression model	Age, sex, diabetes, hypertension, current drinkers, dietary, and lipid-lowering drugs	**1) Ceramides and sphingolipids:** Cer species comprising short-chain fatty acyls (16–18C) and long chain fatty acids. **2) Phospholipids: PC** PC (22:6/13:0), PC (36:6), PC(37:6), PC (38:3), PC (38:5), PC(38:6), PC (38:8), PC (39:3), PC (40:7), PC (40:8), PC (40:9), PC (42:7), PC (42:9), PC (42:10), PC (37:1), PC (38:1); **PE** PE (16:0/22:6), PE (18:0/22:6), PE (18:0p/22:6), PE (18:1p/22:6) and PE (20:0p/22:6) **3) Cholesterol esters and glycerolipids**: ChE (16:0), ChE (17:0), ChE (18:1), ChE (18:1), ChE (20:3), ChE (20:4), and ChE (20:5); DG (12:0/20:5) and DG (18:0/18:0)	**1) Ceramides and sphingolipids:** The level of various Cer species were lower in plasma of VaD patients.SM species did not significantly differ between VaD patients and controls. **2) Phospholipids:** VaD patients had higher levels of plasma LPC. LPC (22:6) showed a significantly higher level in VaD. Major phospholipids classes including PC, PE and PI showed a tendency to be lower in VaD patients compared to healthy controls. **3) Cholesterol esters and glycerolipids:** Most ChEs showed lower abundance in VaD patients
Liu et al. (5)	Principal component analysis (PCA);partial least-squares discriminant analysis (PLS-DA);orthogonal partial least-squares discriminant analysis (OPLS-DA); unit variance (UV); logistic regression model	______	Carnitine, creatinine, glutamine, proline, N-acetylneuraminic acid, hypoxanthine, uric acid, tyrosine, kynurenine, phenylalanine, sphingosine-1-phosphate, and palmitoylcarnitine, citric acid, valine, isoleucine, tryptophan, and lysoPCs	**(1)**The serum samples of stroke patients exhibited higher levels of carnitine, creatinine, glutamine, proline, N-acetylneuraminic acid, hypoxanthine, uric acid, tyrosine, kynurenine, phenyl-alanine, sphingosine-1-phosphate, and palmitoylcarnitine in combination with lower levels of citric acid, valine, isoleucine, tryptophan and lysoPCs. **(2)** Sixteen differential metabolites were identified as potential markers for discriminating between the stroke patients with and without cognitive impairment. In contrast with the PSNCI group, the levels of carnitine, glutamine, uric acid, tyrosine, kynurenine, and phenylalanine were increased in the PSCI group, whereas the levels of valine, isoleucine, tryptophan, lysoPCs, palmitoylcarnitine, and stearoylcarnitine were markedly decreased. **(3)** Three serum metabolites [glutamine, kynurenine, and LysoPC(18:2)] were identified as candidate diagnostic biomarkers for PSCI, and their combined use yielded good diagnostic capacity for PSCI by receiver operating characteristic curves.
Zarrouk et al. (12)	Student's *t*-test; Bonferroni correction; Receiver operating characteristic curves (ROC); Spearman correlation	_______	Fatty acid profiles of red blood cells and plasma, including SFA (sum of saturated fatty acids), MUFA (sum of monounsaturated fatty acids), PUFA (sum of polyunsaturated fatty acids), OA (oleic acid), LA (linoleic acid), ALA (alpha linolenic acid), AA (arachidonic acid), EPA (eicosapentaenoic acid), DHA (docosahexaenoic acid)	Significant variations of several SFAs, including VLCFAs; C26:0 could constitute a convenient peripheral blood biomarker of dementia, including AD.
Zhang et al. (13)	Student's *t*-test; Chi-square test; Logistic regression model; Pearson	_______	Lp-PLA2	The serum Lp-PLA2 in VD group were significantly higher than the non-dementia group, the Lp-PLA2 in VCIND group were significantly higher than the nomal cognition group. The result of Logistic regression showed that Lp-PLA2 was a significant risk factor of VD, and its level was negatively correlated with the score of cognitive function. The cutoff value of Lp-PLA2 between the VD and non-dementia was 164.65 ng/ml and was 120.8 ng/ml between the normal cognition group and VCIND group.
Niu et al. (14)	Logistic regression model; Spearman's correlation	_______	Lp-PLA2	Lp-PLA2 was the independent risk factors for cognitive impairment in ischemic CSVD patients. Serum levels of and Lp-PLA2 were significantly higher in moderate and severe CI groups than in mild CI group and in severe CI group than in moderate CI group and were positively related with the severity of CI in ischemic CSVD patients
Zhang et al. (15)	Student's *t*-test; Chi-square test; Logistic regression model	_______	Lp-PLA2 activity	The activity of Lp-PLA2 was significant higher in VCIND group compared to healthy controls. And the Lp-PLA2 activity increased as MoCA scores went down.
Hou et al. (16)	Student's *t*-test; Chi-square test; Logistic regression model	Age, sex, BMI, blood pressure, blood glucose, and blood fats	Lp-PLA2	Plasma Lp-PLA2 levels in AD group and VaD group were significantly higher than those in control group, and plasma Lp-PLA2 level in VaD group was significantly higher than that in AD group. The elevated plasma Lp-PLA2 level was associated with high risk of AD and VaD, and the correlation with VaD was greater than AD.
Bai et al. (17)	Student's *t*-test; Chi-square test	_______	Lp-PLA2	Plasma Lp-PLA2 levels was significant higher in VCIND group compared to controls.

#### Phospholipid Metabolism

Phospholipid metabolism was significantly associated with VCI. Major glycerophospholipids classes including PC, PE, and PI showed a tendency to be lower in VaD patients than in healthy controls (10). Phatidylcholines lipids containing highly unsaturated FA chains, including PC (22:6/13:0), PC (36:6), PC (37:6), PC (38:3), PC (38:5), PC (38:6), PC (38:8), PC (39:3), PC (40:7), PC (40:8), PC (40:9), PC (42:7), PC (42:9), and PC (42:10), were frequently affected. This suggests that the polyunsaturated FA chain in phospholipids might have a neuroprotective role. With regard to PE lipids, all significant differences were observed in lipids containing a FA chain of 22:6. In addition, VaD patients had higher levels of plasma lysophosphatidylcholines (LPC), especially LPC (22:6) which showed a significantly higher level in VaD (10). However, other group came to the opposite conclusion. LPC, including LPC (14:0), LPC (18:2), LPC (16:0), LPC (18:1), LPC (18:0), and LPC (20:1), significant decline in plasma collected from stroke subjects and a decreased level of LPCs were observed in PSCI group compared to post-stroke no cognitive impairment (PSNCI) group (5). This conflicting finding may be due to the different biospecimen, participants and analytical platforms the two studies selected. Liu's study was undergone in Australian and the lipid analysis were performed by LC ESI-MS/MS. Whereas, Liu used UHPLC-Q-TOFMS to analysis metabolic disorders in Chinese patients. Besides, this may also indicate pleiotropic role of LPCs in the disease process. Thusly, to test these hypothesis, further studies are needed to elucidate the mechanism by targeted plasma lipidomics platform of LPC in VCI patients.

Six studies assessed the association between lipoprotein-associated phospholipase A2 (Lp-PLA2) and VCI, indicating that Lp-PLA2 was a risk factor for VCI. Zhang (15) investigated the activity of Lp-PLA2 and found that the activity of Lp-PLA2 was significantly higher in vascular cognitive impairment no dementia (VCIND) patients than in healthy controls. Lp-PLA2 activity increased as Montreal cognitive assessment (MoCA) scores decreased. Bai found that plasma Lp-PLA2 levels were significantly higher in the VCIND group than in the controls and were also significantly higher in the VaD group than in the VCIND group (17, 18). Moreover, plasma Lp-PLA2 levels in the Alzheimer's disease (AD) group and VaD group were significantly higher than those in the control group, and plasma Lp-PLA2 levels in the VaD group were significantly higher than those in the AD group, which suggested that the elevated plasma Lp-PLA2 levels were associated with a high risk of AD and VaD, and the correlation with VaD was greater than that with AD (16). Similar results were found by Zhang et al. (13) and Niu et al. (14) in the concentration of serum Lp-PLA2. The serum Lp-PLA2 in the VD group was significantly higher than that in the non-dementia group, and the Lp-PLA2 in the VCIND group was significantly higher than that in the normal cognition group. The Logistic regression results showed that Lp-PLA2 was a significant risk factor for VaD, and its level was negatively correlated with cognitive function score. Researchers also reported that the value of Lp-PLA2 between VD and non-dementia was 164.65 ng/ml and was 120.8 ng/ml between the normal cognition group and VCIND group (13).

Phospholipid is a critical component of the plasma membrane and is involved in the trafficking of neural cells and the release of neurotransmitters (19). Phatidylcholines plays a neuroprotective role by maintaining neuronal membrane completeness and providing an essential supply of choline. Increasing evidence suggests that PC is highly predictive of cognitive decline (20). Lower levels of PCs may be due to the abnormal activity of PLA2. PLA2 is a class of enzymes that hydrolyse glycerophospholipids to produce free FAs and LPCs. Emerging evidence has demonstrated that Lp-PLA2 plays a critical anti-atherosclerotic and pro-atherosclerotic role (21). In total, these studies showed impaired phospholipid anabolism in different biospecimens in tandem with increased catabolism of phospholipids due to the excessive activation of PLA2. As an important signaling molecule, LPCs display multiple biological functions, such as involvement in cellular proliferation and regulation of inflammation (22, 23). Lower levels of plasma LPC were demonstrated in patients with AD and mild cognitive impairment (24). However, whether LPCs increase or decrease in VCI patients is uncertain. Additional investigations are needed to elucidate the mechanism and the pleiotropic role of LPCs.

#### Sphingolipid Metabolism

Two studies investigated sphingolipids in the context of VCI. Liu found that Cer, especially Cer species comprising short-chain fatty acyls (16–18C) and long-chain FAs, were significantly lower in VaD (10). Other studies showed that a high level of sphingosine 1-phosphate was observed in stroke subjects relative to healthy controls (5). However, sphingomyelin (SM) species and sphingosine 1-phosphate did not significantly differ between VaD patients and controls (10).

Sphingolipids are not merely essential structural element of cellular membranes but also play prominent roles in regulating cellular events, for instance governing cell proliferation, being involved in the differentiation of neurons and maintaining myelin stability (25). Dysregulation of sphingolipid metabolism has been established in the pathogenesis of several neurological diseases such as AD (26, 27) and Parkinson's disease (28). However, only Cer was significantly different between VCI patients and the control group in our research. Cer is a group of lipids formed by covalently linking sphingosine to a FA. Cer is central in sphingolipid metabolism as it can be converted into more complex sphingolipids, such as GalCer and sphingosine, though different pathways (25). Cer deficiency has been revealed to be associated with WM demyelination in multiple sclerosis (MS) patients (29). Vascular cognitive impairment is likely to share the same pathological process because myelin and axonal loss caused by severe WM changes might play a role in the multiple functional changes that occur in VCI. Thus, Cer might be the potential biomarkers for VCI. Our finding does not draw into conclusion that other sphingolipids are not involved in the disease process because brain and peripheral lipids are two different entities and separated by a strict blood–brain barrier (BBB). Therefore, the lipidomics of CSF or post-mortem brain samples is in need in the future to fully explore sphingolipid-related mechanisms in VCI.

#### Fatty Acids

Amira (12) used gas chromatography with or without MS to investigate the relationship between FAs and dementia. Plasma accumulation of C26:0 and C24:0 was observed in vascular dementia patients compared with a control group of elderly individuals. This study also establishes that C26:0 could constitute a convenient peripheral blood biomarker of dementia.

Very long-chain fatty acids (VLCFAs: C ≥20) including C26:0 are abundant in myelin sphingolipids (30). In the brain, VLCFAs can form complex lipids that can destabilize cell signaling and break cellular membranes. Very long-chain fatty acids are involved in the main function of peroxisomes (31). Studies have shown that peroxisomal dysfunction is involved in AD (32, 33) and the accumulation of VLCFAs was observed in the brains of AD patients (34). In addition, a decreased level of VLCFAs could result from enhanced elongase activities. Therefore, the accumulation of plasma C26:0 in VCI patients could be explained by peroxisomal dysfunctions and enhanced FA elongation.

#### Cholesterol Metabolism

Researchers (10) found that lower levels of most ChEs were associated with VaD, including ChE (16:0), ChE (17:0), ChE (18:1), ChE (18:1), ChE (20:3), ChE (20:4), and ChE (20:5). In plasma, ChE is synthesized by the conversion of FAs to cholesterol from PC, catalyzed by lecithin: cholesterol acyl transferase (LCAT) (35). Hence, the reduction in PC may be linked to dysregulation of specific steps in cholesterol metabolism in VaD.

#### Glycerides Metabolism

Liu (10) also found that DG (12:0/20:5), DG (18:0/18:0), and most triacylglycerols (TGs) showed significantly higher abundance in VaD. However, those containing the FA chain 15:0 in particular were diminished in plasma from VaD patients. DGs are important components for structural integrity and cell signaling. The following metabolic pathways could modulate the levels of DGs: (i) generation of glycerophospholipids; (ii) conversion to phosphatidic acid by DG kinase; (iii) hydrolysis by DG lipase, lipoprotein lipase (LPL), and hormone-sensitive lipase to synthesize monoacylglycerols (MAGs); (iv) acylation of DG to generate TGs (36). Phosphorylation of DG to synthesize phosphatidic acids has been detected to be decreased in the brains of AD patients (37) and the same mechanism might also contribute to the increased levels of plasma DGs in VaD. Since a lower level of PI in VaD was found in Liu's study, glycerophospholipid degradation may also be responsible for the elevations in DG levels in VaD. Additionally, since PLA2 breaks down lysophosphatidylethanolamine (LPE) into DG, the reduction in PE may also play a role in the increased DG levels. Thus, we suppose that the development of VCI will influence the entire homeostasis of lipid metabolism.

## Discussion and Conclusion

Through our comprehensive review, we identified nine original articles that identified the roles of lipids associated with VCI. To the best of our knowledge, this is the first systemic review concerning the relationship of lipid metabolism and VCI to date. We have identified potential lipid metabolism pathways that might be involved in the process. Most importantly, our systematic review revealed that several blood-based metabolites might be proposed as potential biomarkers for VCI, which provides direction for further validation.

**Figure 2:**
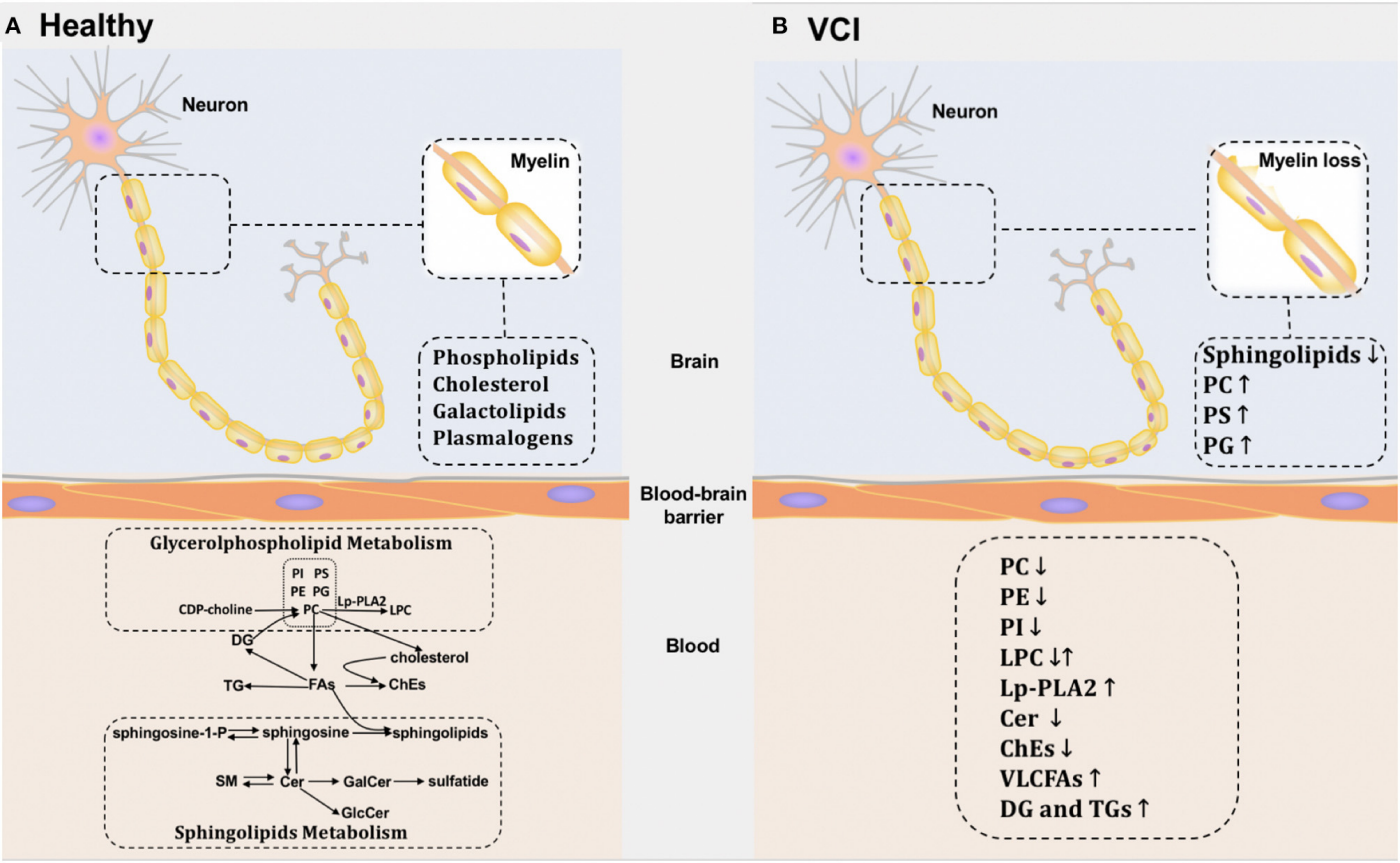
Potential lipid metabolic pathways in brain and blood of healthy individuals and VCI patients. **(A)** The potential metabolic pathways of healthy individuals in brain and blood. **(B)** The potential metabolic pathways of VCI patients in brain and blood.

We revealed that several metabolites are likely to act as potential blood-based biomarkers for VCI, such as PC lipids containing highly unsaturated FA chains, PE lipids containing C22:6, LPC, Lp-PLA2, ChEs, and VLCFAs (especially C26:0). Moving forward, important questions require further investigation: Why does these lipid metabolism disorder occur in VCI patients? Is the lipid change a causative factor or just a consequence of VCI? It is of great importance to address these questions, which might help us better understand the disease pathology (Figure 2). Skrobot revealed that seven pathologies were suggested to contribute to VCI and predict the degree of impairment: large infarcts, lacunar infarcts, microinfarcts, myelin loss, arteriolosclerosis, leptomeningeal cerebral amyloid angiopathy (CAA), and perivascular space dilation (38). Among them, myelin loss and WM changes are likely to have roles in lipid metabolic disorders, since lipids including phospholipids and cholesterol are major constituents of myelin. Researchers verified that WM injuries were related to an increased risk of VCI or the disease itself. They can be detected using MRI, including WMHs, loss of integrity and volume loss (39). White matter injuries are related to myelin loss, but the exact mechanisms and consequences remain unknown and are of great interest to researchers.

Interestingly, changes in similar biomarkers have also been described in post-mortem metabolites, which provides further evidence that lipids might be involved in these pathologies. Overall reductions in sphingolipids were detected in SIVD patients' WM. Increases were observed in numerous phospholipid classes including PC, phosphatidylserine (PS), and PG (40). However, the variation tendency of these metabolites in the CNS appears to be different from our previous observation in the periphery. These conflicting findings may be caused by the BBB, which maintains CNS homeostasis by regulating the transport of solutes between blood and brain (41). The lipids in the brain and periphery are two units that are separated by the BBB. However, the exact mechanism remains unclear. Additionally, blood lipids may also be affected by damage to peripheral organs, which limits the accuracy of any association between changes in blood lipid levels and brain pathology. In order to understand the lipid-related mechanism of VCI, comprehensive lipidomics studies of CSF or post-mortem brains are needed in the future.

Although we thoroughly studied articles on blood-based biomarkers of VCI, it is difficult to reach a consistent conclusion. We failed to extract data and perform meta-analysis or quantitative analysis since related research is limited. All the metabolite targets and their biospecimen are summarized in Table 1 and the Results section. Based on these results, Min Liu indicated that PSCI and PSNCI patients could be well-stratified with high accuracy by using the combination of glutamine, kynurenine, and LPC (18:2). Other analysis demonstrated that Cer and ChE ranked as the top two in the order of importance for classification of VaD. Therefore, several aspects should be taken into consideration. First, since the timescales of our eligible studies are lengthy, the diagnostic criteria of VCI have changed so far. Thus, we not just included studies with VCI patients, the participants of the included studies varied from VCIND, PSCI, and VCI to VaD. This difference among articles introduce heterogeneity. For example, one study examined samples of stroke victims (5). Although it indicated that PSCI increased the severity of metabolic disturbance or had different potential pathways compared with stroke, stroke itself could lead to different lipid metabolism and might introduce bias. Thusly, these results need to be further revealed in the larger concept of VCI. Additionally, large heterogeneity in study design, populations, cognitive testing was noted. Second, diverse metabolomics analytical platforms and different biological samples (plasma or serum) resulted in variations in the data. Less than half of the studies used lipidomics platforms, which failed to build full vision of the disease process. Besides, different analytical techniques are of different accuracies. Therefore, more non-targeted and targeted metabolomic and lipidomic analyses should be done in the future. Third, differences in statistical approaches could be a further source of inconsistency. Finally, as seven of the nine included studies were from China, there may be some geographical or population-base limitations. Caution must be taken when generalization of the results to the whole population and more studies should be done in other counties. However, the two studies from Australia and Tunesia were not isolated from the other studies in Chinese population. Since all participants from the included studies shared similar age range, these nine studies were statically relevant. Taken together, the evidence suggests a need to standardize the workflow for study design, participant selection, sample collection and storage, and experimental and data procedures. The application of combined analytical platforms is an attractive prospect for future studies. Moreover, we are likely to find a combination of several metabolites instead of one biomarker as a potential indicator for VCI.

To conclude, our study opens new ways to identify biomarkers of VCI. Blood-based samples are relatively convenient and fast to obtain compared to traditional biomarkers. Assuming we could find a suitable marker, we believe that it will be helpful in screening in large-scale clinical trials and for future clinical use. Moving forward, since most of the studies we concluded are cross-sectional studies, we fail to distinguish diagnostic biomarkers from monitoring biomarkers. Thus, longitudinal studies with long follow-up are needed to prospectively validate which biomarkers could be used as monitoring biomarkers in the future. In addition, systematic profiling of the comprehensive brain and peripheral lipidome of VCI patients is still lacking and therefore requires comprehensive non-targeted analyses. Based on the results of non-targeted analyses, targeted quantitative metabolomic and lipidomic analyses should be performed to determine possible biomarkers, which will improve our understanding of the disease.

## Data Availability Statement

The original contributions presented in the study are included in the article/Supplementary Material, further inquiries can be directed to the corresponding author.

## Author Contributions

QQ, YY, and YX searched and acquired data. QQ and YY drafted the manuscript. FW and YT revised the article. XW and YW helped to draft the manuscript. All authors read and approved the final manuscript.

## Funding

This work was supported by Capital's Funds for Health Improvement and Research (grant number CFH 2020-4-1033).

## Conflict of Interest

The authors declare that the research was conducted in the absence of any commercial or financial relationships that could be construed as a potential conflict of interest.

## Publisher's Note

All claims expressed in this article are solely those of the authors and do not necessarily represent those of their affiliated organizations, or those of the publisher, the editors and the reviewers. Any product that may be evaluated in this article, or claim that may be made by its manufacturer, is not guaranteed or endorsed by the publisher.

## Abbreviations

VCI: vascular cognitive impairment
VaD: vascular dementia
SIVD: subcortical ischaemic vascular disease
PSCI: post-stroke cognitive impairment
CNS: central nervous system
WM: white matter
CSF: cerebrospinal fluid
MS: mass spectrometry
NMR: nuclear magnetic resonance spectroscopy
RCT: randomized controlled trial
MRS: magnetic resonance spectroscopy
UHPLC-Q-TOFMS: ultra-high performance liquid chromatography-quadrupole time-of-flight mass spectrometer
GC/MS-SIM: gas chromatograph/mass spectrometry-selected-ion monitoring
LC: liquid chromatograph
PC: phosphatidylcholine
PE: phosphatidylethanolamine
Cer: ceramide
ChE: cholesterol ester
DG: diacylglycerol
PI: phosphatidylinositol
LPC: lysophosphatidylcholines
PSNCI: post-stroke no cognitive impairment
Lp-PLA2: lipoprotein-associated phospholipase A2
VCIND: vascular cognitive impairment no dementia
MoCA: Montreal cognitive assessment
AD: Alzheimer's disease
SM: sphingomyelin
MS: multiple sclerosis
BBB: blood-brain barrier
VLCFAs: very long-chain fatty acids
LCAT: cholesterol acyl transferase
TG: triacylglycerols
LPE: lysophosphatidylethanolamine
MAGs: monoacylglycerols
PS: phosphatidylserine
PG: phosphatidylglycerol.
