# Supplementary material for: Lipid Metabolism in the Development and Progression of Vascular Cognitive Impairment: A Systematic Review
Source: Front Neurol. 2021 Nov 19;12:709134. doi: 10.3389/fneur.2021.709134 (PMC8639494; doi:10.3389/fneur.2021.709134)
Supplement: Supplementary file 1 [file Data_Sheet_1.docx]

**Supplementary Table 1. Quality Assessment of cross-sectional study**

| Reference | Define the source of information (survey, record review) | List inclusion and exclusion criteria for exposed and unexposed subjects (cases and controls) or refer to previous publications | Indicate time period used for identifying patients | Indicate whether or not subjects were consecutive if not population-based | Indicate if evaluators of subjective components of study were masked to other aspects of the status of the participants | Describe any assessments undertaken for quality assurance purposes (e.g, test/retest of primary outcome measurements) | Explain any patient exclusions from analysis | Describe how confounding was assessed and/or controlled | If applicable, explain how missing data were handled in the analysis | Summarize patient response rates and completeness of data collection | Clarify what follow-up, if any, was expected and the percentage of patients for which incomplete data or follow-up was obtained | Summed scores |
| --- | --- | --- | --- | --- | --- | --- | --- | --- | --- | --- | --- | --- |
| (Y.Liu et al.,2020) | 1 | 1 | 1 | 1 | 1 | 1 | 0 | 1 | 0 | 1 | 0 | 8 |
| (M.Liu et al.,2015) | 1 | 1 | 1 | 0 | 1 | 1 | 0 | 0 | 0 | 0 | 0 | 5 |
| (Zarrouk et al., 2015) | 1 | 1 | 1 | 1 | 1 | 1 | 0 | 0 | 0 | 1 | 0 | 7 |
| (Niu et al., 2020) | 1 | 1 | 1 | 1 | 1 | 1 | 0 | 1 | 0 | 1 | 0 | 8 |
| (Hou et al., 2018) | 1 | 1 | 1 | 1 | 1 | 1 | 0 | 1 | 0 | 0 | 0 | 7 |
| (Bai et al., 2014) | 1 | 1 | 1 | 1 | 1 | 1 | 0 | 0 | 0 | 0 | 0 | 6 |
| (Bai & Su, 2010) | 1 | 1 | 1 | 1 | 1 | 1 | 0 | 0 | 0 | 0 | 0 | 6 |

**Supplementary Table 2. Quality Assessment of case-control study**

| Reference | Selection, including adequate definition of cases, representativeness of cases, and selection and definition of controls (1 point per criterion); | Comparability, 2 points if the analysis was adjusted for most potential confounders, and 1 point if the analysis was only adjusted for age and sex (or age and sex matched) | Exposure, including assessment of exposure, and same method of ascertainment for cases and controls (1 point per criterion) | Summed scores |
| --- | --- | --- | --- | --- |
| (Q. Zhang et al., 2019) | 4 | 2 | 3 | 9 |
| (H. Zhang et al., 2010) | 3 | 0 | 3 | 6 |
